# Obese patients have higher rates of polymicrobial and Gram-negative early periprosthetic joint infections of the hip than non-obese patients

**DOI:** 10.1371/journal.pone.0215035

**Published:** 2019-04-08

**Authors:** Claudia A. M. Löwik, Wierd P. Zijlstra, Bas A. S. Knobben, Joris J. W. Ploegmakers, Baukje Dijkstra, Astrid J. de Vries, Greetje A. Kampinga, Glen Mithoe, Aziz Al Moujahid, Paul C. Jutte, Marjan Wouthuyzen-Bakker, for the Northern Infection Network Joint Arthroplasty (NINJA)

**Affiliations:** 1 Department of Orthopaedic Surgery, University of Groningen, University Medical Center Groningen, Groningen, The Netherlands; 2 Department of Orthopaedic Surgery, Medical Center Leeuwarden, Leeuwarden, The Netherlands; 3 Department of Orthopaedic Surgery, Martini Hospital, Groningen, The Netherlands; 4 Department of Medical Microbiology and Infection Prevention, University of Groningen, University Medical Center Groningen, Groningen, The Netherlands; 5 Department of Medical Microbiology, Certe Medical Diagnostics and Advice, Groningen, the Netherlands; 6 Center for Infectious Diseases Friesland, Izore, Leeuwarden, the Netherlands; Universitatsklinikum Hamburg-Eppendorf, GERMANY

## Abstract

**Background:**

Obese patients are more likely to develop periprosthetic joint infection (PJI) after primary total joint arthroplasty. This study compared the clinical and microbiological characteristics of non-obese, obese and severely obese patients with early PJI, in order to ultimately optimize antibiotic prophylaxis and other prevention measures for this specific patient category.

**Methods:**

We retrospectively evaluated patients with early PJI of the hip and knee treated with debridement, antibiotics and implant retention (DAIR) between 2006 and 2016 in three Dutch hospitals. Only patients with primary arthroplasties indicated for osteoarthritis were included. Early PJI was defined as an infection that developed within 90 days after index surgery. Obesity was defined as a BMI ≥30kg/m^2^ and severe obesity as a BMI ≥35kg/m^2^.

**Results:**

A total of 237 patients were analyzed, including 64 obese patients (27.0%) and 62 severely obese patients (26.2%). Compared with non-obese patients, obese patients had higher rates of polymicrobial infections (60.3% vs 33.3%, p<0.001) with more often involvement of *Enterococcus* species (27.0% vs 11.7%, p = 0.003). Moreover, severely obese patients had more Gram-negative infections, especially with *Proteus* species (12.9% vs 2.3%, p = 0.001). These results were only found in periprosthetic hip infections, comprising Gram-negative PJIs in 34.2% of severely obese patients compared with 24.7% in obese patients and 12.7% in non-obese patients (p = 0.018).

**Conclusions:**

Our results demonstrate that obese patients with early periprosthetic hip infections have higher rates of polymicrobial infections with enterococci and Gram-negative rods, which stresses the importance of improving preventive strategies in this specific patient category, by adjusting antibiotic prophylaxis regimens, improving disinfection strategies and optimizing postoperative wound care.

## Introduction

Obesity is a major health concern worldwide, as obesity has nearly tripled in the last decades. Currently, 39% of adults are overweight and 13% are obese [1]. Obesity is not only associated with an increased risk of comorbidities, such as hypertension, diabetes mellitus and ischaemic heart disease [2], but due to early development of osteoarthritis of weight bearing joints, obese patients are also more likely to receive joint arthroplasty [3]. Implantation of joint arthroplasties may lead to postoperative complications, particularly in obese patients [4]. The most important complication is periprosthetic joint infection (PJI), occurring in approximately 1–2% of patients receiving joint arthroplasty [5]. PJI has a large impact on patient’s quality of life and is accompanied by high healthcare costs. Literature indicates that the risk of PJI increases exponentially with the body mass index (BMI): i.e. severely obese patients have a four times increased risk of PJI compared with non-obese patients [6, 7].

There are many hypotheses for the increased risk of PJI in obese patients [8]. First of all, obese patients are prone to diminished wound healing because of increased surface tension at the surgical site and prolonged wound leakage due to bulky subcutaneous tissue [9]. Secondly, higher glucose levels in obese patients with diabetes mellitus increase the risk of infection [10]. Thirdly, the applied cefazolin dosage as antibiotic prophylaxis may not be sufficient to achieve adequate tissue concentrations in obese patients, especially in patients weighing more than 120kg or with a BMI >40kg/m^2^ [11–13]. Finally, obese patients have increased bacterial colonization of the skin compared with non-obese patients, particularly in the groin [14, 15]. Currently applied antibiotic prophylaxis may not provide full coverage for these microorganisms.

To optimize prevention measures for obese patients receiving primary total joint arthroplasty, we aimed to describe the clinical and microbiological characteristics of early PJI in obese and severely obese patients.

## Patients and methods

### Study design

We retrospectively reviewed patients with early PJI of the hip and knee who were treated with debridement, antibiotics and implant retention (DAIR) in one university hospital (University Medical Center Groningen) and two general hospitals (Martini Hospital and Medical Center Leeuwarden) between January 2006 and December 2016. PJI was diagnosed according to the diagnostic criteria defined by the Musculoskeletal Infection Society (MSIS) [16]. In case of a single positive culture with a highly virulent microorganism, PJI was diagnosed in consultation with the infectious diseases specialist or medical microbiologist. Early PJI was defined as an infection developed within 90 days after index surgery [17]. Only patients with primary arthroplasties indicated for osteoarthritis were included. Patients who underwent arthroscopic debridement or did not meet the MSIS criteria were excluded.

The following clinical variables were collected: sex, age, BMI, comorbidities, medication, inflammatory markers and specifications of index surgery and the DAIR procedure. BMI was measured during the preoperative anesthetic screening prior to joint arthroplasty surgery. Patients were categorized according to BMI, with a cut-off value of 30kg/m^2^ (i.e. class 1 obesity) and 35kg/m^2^ (i.e. class 2 obesity / severe obesity) [1, 18]. Early failure was defined as the need for 1) a second DAIR procedure, 2) revision arthroplasty or definitive implant removal, 3) infection-related death or 4) suppressive antimicrobial treatment, all within 60 days after initial debridement. Overall failure was defined as infection-related death or the need for implant removal at any time point after initial debridement.

The following microbiological variables were collected: number of positive cultures, type of microorganism and resistance patterns of cultured microorganisms.

### Index surgery and DAIR procedure

Prior to joint arthroplasty patients performed a whole-body cleansing with chlorhexidine scrub. In case of abundant hair growth on the knee or groin region, hair was removed from the surgical site by use of a clipper. Prior to surgical incision, the skin was disinfected with a solution of povidone tincture in 75% alcohol. In case patients were allergic to povidone, the skin was disinfected by a chlorhexidine-alcohol solution. Cefazolin was administered as antibiotic prophylaxis 30 to 60 minutes prior to incision. According to local protocol, cefazolin dosage was one gram for patients <80kg and two grams for patients ≥80kg. In all patients receiving total hip arthroplasty the posterolateral approach was used. Total knee arthroplasties were inserted via the medial parapatellar approach. The incisions were closed with staples and dressed with absorbent bandages. In case of wound leakage in the postoperative period a pressure bandage was applied.

DAIR procedure consisted of opening the wound via the pre-existing incision, excising hematoma and avital tissue and irrigating the wound thoroughly with three to six liters of saline. Modular components were exchanged and optionally gentamicin-impregnated beads or sponges were inserted into the joint cavity according to local protocol and clinical judgement of the operating orthopaedic surgeon. Multiple cultures of deep tissue and synovial fluid were obtained during debridement. Cefuroxime was started as empirical intravenous antimicrobial treatment after obtaining cultures and was adjusted according to the culture results. Intravenous antimicrobial treatment was continued for at least two weeks before switching to an oral regimen, which was continued for an additional ten weeks. Rifampin was added to the antimicrobial treatment regimen in infections caused by rifampin-susceptible staphylococci.

### Statistical analysis

Categorical variables were presented in absolute frequencies and percentages. Continuous variables were expressed as mean and standard deviation (SD) or as median and interquartile range (IQR) when not normally distributed according to the Kolmogorov-Smirnov test of normality. The statistical comparison of categorical variables was performed using the Chi-square test. Parametric continuous variables were compared using the Student’s t-test and one-way ANOVA. Non-parametric continuous variables were compared with the Mann-Whitney U test. Statistical significance was defined as a two-tailed p <0.05. Statistical assessment was carried out with IBM SPSS Statistics (version 24.0, Chicago, USA).

### Ethical review committee statement

The Institutional Review Boards of the University Medical Center Groningen, Martini Hospital and Medical Center Leeuwarden approved this study. Each Institutional Review Board approved a waiver for obtaining written informed consent from the participants, since this was an observational study in which the data were analyzed anonymously. The study is conducted in accordance to the ethical standards in the 1964 Declaration of Helsinki, the Medical Research Involving Human Subjects Act (WMO) and the Good Clinical Practice standard (GCP).

## Results

### Clinical characteristics

A total of 356 patients with early PJI of the knee or hip treated with DAIR were identified. We excluded 52 patients with revision arthroplasties and 67 patients with primary arthroplasties placed for other indications than osteoarthritis (fracture (n = 62), rheumatoid arthritis (n = 2) and malignancy (n = 3)). A total of 237 included patients had a mean age of 71.3 years (SD 10.5, range 27–91 years) and 63.7% of patients (n = 151) were female. 160 patients (67.5%) had a periprosthetic hip infection and 77 patients (32.5%) had a periprosthetic knee infection. Patients experienced a mean duration of symptoms of infection of 6.8 days (SD 5.9, range 1–21 days) before DAIR procedure. DAIR procedure was performed at a mean of 20.7 days (SD 11.7, range 7–74 days) after index surgery. The median follow-up was 1.7 years (IQR 1.0–3.3) and was similar in obese and non-obese patients (1.8 years (IQR 1.0–3.1) vs 1.5 years (IQR 1.0–3.4), p = 0.732).

From the included cohort (n = 237) a total of 126 patients (53.2%) were obese. Of these obese patients 62 were severely obese (49.2%). Non-obese, obese and severely obese patients were of similar age (72.3 years vs 70.5 years vs 69.4 years, p = 0.229), while severely obese patients were more often of female gender than non-severely obese patients (74.2% vs 60.0%, p = 0.046). Regarding comorbidities, both obese and severely obese patients had a higher incidence of hypertension compared with non-obese patients and non-severely obese patients respectively (69.0% vs 56.8%, p = 0.050 and 75.8% vs 58.9%, p = 0.017), while diabetes mellitus only had a higher incidence in severely obese patients (35.5% vs 17.1%, p = 0.003). Regarding symptoms at initial DAIR, severely obese patients had a higher incidence of redness of the wound compared with non-severely obese patients (39.4% vs 56.5%, p = 0.020). There was a trend towards more wound leakage in obese patients, but this difference was not statistically significant (82.0% vs 89.7%, p = 0.088) (Table 1).

**Table 1:** Clinical characteristics of early PJIs according to BMI.

Variables		BMI <30 (n = 111)	BMI ≥30 (n = 126)	P value	BMI <35 (n = 175)	BMI ≥35 (n = 62)	P value
Sex	Male	43 (38.7%)	43 (34.1%)	.461	70 (40.0%)	16 (25.8%)	**.046**
Age in years	Mean (SD)	72.3 (11.7)	70.5 (9.2)	.192	72.0 (10.8)	69.4 (9.3)	.098
Comorbidities	Diabetes Mellitus	20 (18.0%)	32 (25.4%)	.171	30 (17.1%)	22 (35.5%)	**.003**
	Hypertension	63 (56.8%)	87 (69.0%)	.050	103 (58.9%)	47 (75.8%)	**.017**
	Ischaemic heart disease	17 (15.3%)	23 (18.3%)	.547	33 (18.9%)	7 (11.3%)	.172
	COPD	17 (15.3%)	28 (22.2%)	.176	30 (17.1%)	15 (24.2%)	.224
Medication	Anticoagulants	24 (21.6%)	24 (19.0%)	.623	36 (20.6%)	12 (19.4%)	.838
	Corticosteroids	18 (16.2%)	10 (7.9%)	**.049**	22 (12.6%)	6 (9.7%)	.544
Smoker		19 (18.3%)	18 (15.0%)	.511	28 (17.1%)	9 (15.0%)	.711
Serum inflammatory markers	Leukocyte count, x 10^9^/L	12.3 (5.5)	10.4 (3.8)	**.002**	11.4 (5.0)	10.8 (3.9)	.392
	CRP, mg/L	92.9 (96.1)	83.1 (98.9)	.439	86.7 (94.7)	90.4 (106.0)	.797
Symptoms	Wound leakage	91 (82.0%)	113 (89.7%)	.088	148 (84.6%)	56 (90.3%)	.261
	Redness	44 (39.6%)	60 (47.6%)	.217	69 (39.4%)	35 (56.5%)	**.020**
	Fever	25 (22.5%)	21 (16.7%)	.255	33 (18.9%)	13 (21.0%)	.718
Joint	Hip	79 (71.8%)	81 (64.8%)	.342	122 (70.5%)	38 (61.3%)	.473
	Knee	32 (28.2%)	45 (35.2%)		51 (29.5%)	24 (38.7%)	
Cementation	No cement	24 (21.6%)	21 (16.7%)	.354	37 (21.1%)	8 (12.9%)	.295
	Cement without antibiotics	4 (3.6%)	2 (1.6%)		5 (2.9%)	1 (1.6%)	
	Cement with antibiotics	83 (74.8%)	103 (81.7%)		133 (76.0%)	53 (85.5%)	
Drain	No drain	25 (22.9%)	32 (25.6%)	.065	41 (23.7%)	16 (26.2%)	.906
	1 drain	38 (34.9%)	58 (46.4%)		71 (41.0%)	25 (41.0%)	
	2 drains	46 (42.2%)	35 (28.0%)		61 (35.3%)	20 (32.8%)	
Number of DAIRs	Mean (SD)	1.28 (0.49)	1.31 (0.48)	.632	1.29 (0.48)	1.31 (0.50)	.834
Exchange of components		29 (26.4%)	32 (25.4%)	.866	49 (28.2%)	12 (19.4%)	.174
DAIR failure	Early failure	34 (30.6%)	42 (33.3%)	.656	57 (32.6%)	19 (30.6%)	.780
	Overall failure	10 (9.0%)	13 (10.3%)	.734	17 (9.7%)	6 (9.7%)	.993

Bold indicates statistically significant differences. BMI: body mass index; COPD: chronic obstructive pulmonary disease; CRP: C-reactive protein; DAIR: debridement, antibiotics and implant retention; SD: standard deviation

Comparing the variables of the joint replacement surgery and DAIR procedure we did not find any differences between non-obese, obese and severely obese patients (Table 1). Regarding the outcome after DAIR, rates of early failure and overall failure were comparable between non-obese, obese and severely obese patients (30.6% vs 33.3% vs 30.6%, p = 0.740 and 9.0% vs 10.3% vs 9.7%, p = 0.961 respectively). In the majority of patients (n = 67, 88.2%) early failure was due to the need for a second DAIR, five patients (6.6%) needed revision surgery and two patients (2.6%) started with suppressive antimicrobial treatment after initial debridement.

### Microbiological characteristics

Overall, early PJIs were polymicrobial in 113 patients (47.7%). Predominant microorganisms causing the infection were *Staphylococcus aureus* (44.3%), *Staphylococcus epidermidis* (34.6%), *Streptococcus* species (21.5%) and *Enterococcus* species (19.8%). Regarding polymicrobial infections, 66.4% solely involved Gram-positive microorganisms (n = 75), 2.7% solely Gram-negative microorganisms (n = 3), and 31.0% involved both Gram-positive and Gram-negative microorganisms (n = 35). In patients with polymicrobial infections, each obtained culture was positive in 86.7% of cases (n = 98). The mean number of species cultured in these polymicrobial infections was 2.64 (range 2–7), with 2 species being cultured in 56.6% (n = 64), 3 species in 30.1% (n = 34), 4 species in 8.0% (n = 9), 5 species in 4.4% (n = 5) and 7 species in 0.9% (n = 1). In case of a polymicrobial infection, *Staphylococcus epidermidis* was involved in 46.0% of cases, *Staphylococcus aureus* in 41.6%, *Enterococcus* species in 38.1%, *Streptococcus* species in 29.2% and *Proteus* species in 10.6%. The most common combination of microorganisms was *Staphylococcus aureus* together with *Streptococcus* species (n = 7) and *Staphylococcus epidermidis* together with *Enterococcus* species (n = 6).

Obese patients had a higher rate of polymicrobial infections than non-obese patients (60.3% vs 33.3%, p<0.001), with higher prevalence of *Enterococcus* species (27.0% vs 11.7%, p = 0.003). The same applies to severely obese patients compared with non-severely obese patients (polymicrobial infections in 59.7% vs 43.4%, p = 0.028 and *Enterococcus* species in 32.2% vs 15.4%, p = 0.004). Overall, there were no differences regarding Gram-negative microorganisms between obese and non-obese patients, but there were significantly more infections caused by *Proteus* species in severely obese patients compared with non-severely obese patients (12.9% vs 2.3%, p = 0.001).

Analyzing patients with PJI of the hip and knee separately, we found that the higher rate of polymicrobial infections in obese patients, including *Enterococcus* species and Gram-negative microorganisms, was only significant in patients with periprosthetic hip infections (Table 2). Gram-negative rods were isolated in 34.2% of severely obese patients with hip PJI, compared with 24.7% in obese patients and 12.7% in non-obese patients (p = 0.018). *Proteus* species were the main isolated Gram-negative microorganism, with a higher rate in severely obese patients compared with non-severely obese patients (18.4% vs 1.6%, p<0.001). Moreover, *Morganella morganii* was more often involved in severely obese patients (7.9% vs 0.0%, p = 0.002). These differences were not significant in obese patients compared with non-obese patients. Assessment of the microbiological characteristics in patients with periprosthetic knee infections only showed a significant higher rate of involvement of *Enterococcus faecium* in severely obese compared with non-severely obese patients (8.3% vs 0.0%, p = 0.037). There were no other significant differences between non-obese, obese and severely obese patients (Table 3).

**Table 2:** Isolated microorganisms during DAIR in early PJIs of the hip according to BMI.

Variables				BMI <30 (n = 79)	BMI ≥30 (n = 81)	P value	BMI <35 (n = 122)	BMI ≥35 (n = 38)	P value
Percentage of positive cultures		Mean (SD)		91.4 (20.1)	91.8 (18.5)	.917	90.7 (20.9)	94.4 (12.2)	.304
Polymicrobial infection				27 (34.2%)	55 (67.9%)	**<0.001**	56 (45.9%)	26 (68.4%)	**.015**
Gram-positive microorganisms				76 (96.2%)	78 (96.3%)	.975	117 (95.9%)	37 (97.4%)	.678
		*Staphylococcus aureus*		33 (41.8%)	32 (39.5%)	.770	51 (41.8%)	14 (36.8%)	.587
		*Staphylococcus epidermidis*		25 (31.6%)	35 (43.2%)	.131	43 (35.2%)	17 (44.7%)	.291
		*Corynebacterium* species		7 (8.9%)	20 (24.7%)	**.008**	22 (18.0%)	5 (13.2%)	.484
		*Enterococcus* species		10 (12.7%)	24 (29.6%)	**.009**	20 (16.4%)	14 (36.8%)	**.007**
			*Enterococcus faecalis*	7 (8.9%)	20 (24.7%)	**.008**	16 (13.1%)	11 (28.9%)	**.023**
			*Enterococcus faecium*	1 (1.3%)	0 (0.0%)	.310	1 (0.8%)	0 (0.0%)	.576
		*Streptococcus* species		20 (25.3%)	16 (19.8%)	.399	32 (26.2%)	4 (10.5%)	**.043**
		Other gram-positives^a^		3 (3.8%)	3 (3.7%)	.975	4 (3.3%)	2 (5.3%)	.574
Gram-negative microorganisms				10 (12.7%)	20 (24.7%)	.051	17 (13.9%)	13 (34.2%)	**.005**
	*Enterobacteriaceae*	*Escherichia coli*		2 (2.5%)	3 (3.7%)	.670	3 (2.5%)	2 (5.3%)	.386
		*Enterobacter cloacae*		2 (2.5%)	3 (3.7%)	.670	3 (2.5%)	2 (5.3%)	.386
		*Proteus* species^b^		2 (2.5%)	7 (8.6%)	.094	2 (1.6%)	7 (18.4%)	**< .001**
		*Klebsiella* species		1 (1.3%)	0 (0.0%)	.310	1 (0.8%)	0 (0.0%)	.576
		*Morganella morganii*		0 (0.0%)	3 (3.7%)	.084	0 (0.0%)	3 (7.9%)	**.002**
	Non-fermenters	*Pseudomonas* species		3 (3.8%)	3 (3.7%)	.975	5 (4.1%)	1 (2.6%)	.678
		*Acinetobacter* species		1 (1.3%)	2 (2.5%)	.575	2 (1.6%)	1 (2.6%)	.694
Anaerobe microorganisms				3 (3.8%)	4 (4.9%)	.724	4 (3.3%)	3 (7.9%)	.224
*Candida* species				0 (0.0%)	1 (1.2%)	.322	0 (0.0%)	1 (2.6%)	.072

Bold indicates statistically significant differences. BMI: body mass index; SD: standard deviation.

^a^ *Micrococcus luteus* (n = 2), *Microbacterium flavescens* (n = 1), *Dolosigranulum pigrum* (n = 1), *Kocuria* species (n = 1), *Rothia mucilaginosa* (n = 1).

^b^ 8/9 *Proteus* species that were isolated were *Proteus mirabilis*, 1/9 was *Proteus vulgaris*.

**Table 3:** Isolated microorganisms in early PJIs of the knee according to BMI.

Variables				BMI <30 (n = 31)	BMI ≥30 (n = 44)	P value	BMI <35 (n = 51)	BMI ≥35 (n = 24)	P value
Percentage of positive cultures		Mean (SD)		85.3 (30.1)	91.3 (22.0)	.318	86.5 (28.3)	93.8 (18.3)	.251
Polymicrobial infection				9 (29.0%)	20 (45.5%)	.150	18 (35.3%)	11 (45.8%)	.382
Gram-positive microorganisms				27 (87.1%)	42 (95.5%)	.189	46 (90.2%)	23 (95.8%)	.401
		*Staphylococcus aureus*		15 (48.4%)	24 (54.5%)	.599	26 (51.0%)	13 (54.2%)	.797
		*Staphylococcus epidermidis*		8 (25.8%)	14 (31.8%)	.573	15 (25.5%)	9 (37.5%)	.287
		*Corynebacterium* species		3 (9.7%)	5 (11.4%)	.816	5 (9.8%)	3 (12.5%)	.724
		*Enterococcus* species		3 (9.7%)	10 (22.7%)	.142	7 (13.7%)	6 (25.0%)	.229
			*Enterococcus faecalis*	2 (6.5%)	5 (11.4%)	.471	5 (9.8%)	2 (8.3%)	.838
			*Enterococcus faecium*	0 (0.0%)	2 (4.5%)	.229	0 (0.0%)	2 (8.3%)	**.037**
		*Streptococcus* species		6 (19.4%)	9 (20.5%)	.907	12 (23.5%)	3 (12.5%)	.265
		Other gram-positives^a^		1 (3.2%)	2 (4.5%)	.774	2 (3.9%)	1 (4.2%)	.960
Gram-negative microorganisms				4 (12.9%)	7 (15.9%)	.717	8 (15.7%)	3 (12.5%)	.716
	*Enterobacteriaceae*	*Escherichia coli*		0 (0.0%)	1 (2.3%)	.398	1 (2.0%)	0 (0.0%)	.490
		*Enterobacter cloacae*		2 (6.5%)	2 (4.5%)	.718	3 (5.9%)	1 (4.2%)	.758
		*Proteus mirabilis*		1 (3.2%)	2 (4.5%)	.774	2 (3.9%)	1 (4.2%)	.960
		*Klebsiella* species		0 (0.0%)	0 (0.0%)	1.000	0 (0.0%)	0 (0.0%)	1.000
		*Morganella morganii*		0 (0.0%)	0 (0.0%)	1.000	0 (0.0%)	0 (0.0%)	1.000
	Non-fermenters	*Pseudomonas* species		1 (3.2%)	1 (2.3%)	.801	1 (2.0%)	1 (4.2%)	.580
		*Acinetobacter* species		0 (0.0%)	1 (2.3%)	.398	1 (2.0%)	0 (0.0%)	.490
Anaerobe microorganisms				0 (0.0%)	1 (2.3%)	.398	1 (2.0%)	0 (0.0%)	.490
*Candida* species				0 (0.0%)	0 (0.0%)	1.000	0 (0.0%)	0 (0.0%)	1.000

Bold indicates statistically significant differences. BMI: body mass index; SD: standard deviation.

^a^ *Granulicatella adiacens* (n = 2), *Staphylococcus lugdunensis* (n = 1)

Regarding the susceptibility analyses of cefazolin and cefuroxime in Gram-negative microorganisms, results of these tests were only partially available. Microorganisms that are intrinsically resistant to cefazolin and cefuroxime (such as *Enterococcus* species, *Enterobacter cloacae*, *Proteus vulgaris* and *Morganella morganii*) are not indicated. *E*. *coli* was sensitive to cefazolin in all tested cases (n = 3). *Proteus mirabilis* showed intermediate resistance to cefazolin in 33.3% (n = 3). Regarding cefuroxime, *E*. *coli* (n = 6), *Klebsiella* species (n = 1) and *Proteus mirabilis* (n = 11) all showed 100% sensitivity.

## Discussion

This study describes the differences in clinical and microbiological characteristics of early PJI between non-obese, obese and severely obese patients. Our results demonstrated that obese and severely obese patients with periprosthetic hip infections had higher rates of polymicrobial infections with involvement of *Enterococcus* species. Moreover, severely obese patients with PJI of the hip had a higher rate of infections with Gram-negative microorganisms than non-severely obese patients. Interestingly, these results were not found in obese patients with periprosthetic knee infections, which indicates that infections of the hip and knee should be perceived as two separate entities and should therefore be approached differently. Even though the outcome after DAIR was comparable between non-obese, obese and severely obese patients, the differences in microbiological profile between obese and non-obese patients indicate that preventive measures should be adapted for hip arthroplasty surgery in obese patients.

The concept of abundant colonization with multiple microorganisms in the hip region [14, 15] is expected to mainly apply to direct anterior incisions, as previously described in literature [19, 20]. Interestingly, in our cohort obese patients showed abundant colonization with multiple microorganisms as well, even though posterolateral incisions were used for implantation of the hip arthroplasties. Contamination of the wound can occur primarily during surgery or secondarily in the postoperative period. Primary contamination may occur if inadequate antibiotic prophylaxis is applied (either by insufficient dosage or inadequate type of antibiotics) or if the hip region is not thoroughly disinfected. The latter may be prevented by more thorough disinfection of the hip region, for example by using chlorhexidine-alcohol as a local disinfectant. Even though there are no large trials comparing chlorhexidine-alcohol with povidone tincture in 75% alcohol, it is proven that chlorhexidine-alcohol is more effective than povidone-iodine in reducing postoperative infections [21]. This superior protection is mainly attributed to a reduction in Gram-positive skin flora by rapid action and persistent activity despite exposure to bodily fluids [21]. Nevertheless, there was a high number of polymicrobial infections in our cohort, caused by microorganisms susceptible to the local disinfectant as well as the antimicrobial prophylaxis. This may indicate that either the antibiotic dosage was too low or that a significant proportion of PJIs developed due to secondary contamination in the postoperative period, maybe due to prolonged wound leakage, via the retrograde pathway [22]. Although our data showed a trend towards a higher rate of wound leakage in obese patients, this difference was not statistically significant. To prevent secondary contamination, it is important to provide adequate wound care with sterile absorbent dressings and pressure bandages [23]. Therefore, obese patients should be evaluated more extensively after joint arthroplasty, to detect wound complications and provide adequate wound care at an early time point.

A different option for improving preventive measures in obese patients receiving total hip arthroplasty is to increase the dosage of cefazolin, especially since previous studies showed that the currently applied cefazolin dosage as antibiotic prophylaxis may not be sufficient to achieve adequate tissue concentrations in obese patients [11–13]. Another reason to increase the dosage of cefazolin is the higher rate of infections caused by *Proteus* species in obese patients in our cohort, since *Proteus* species have a higher minimum inhibitory concentration for cefazolin than other Enterobacteriaceae [24]. Recently the Dutch guideline for antibiotic prophylaxis was adjusted, in which it is now advised to administer three grams of cefazolin in patients with a BMI >40 kg/m^2^ [25], although our results suggest that this higher dosage may also be beneficial for patients with a BMI >35 kg/m^2^.

Another option is to broaden the antibiotic prophylaxis during total hip arthroplasty surgery in obese patients, especially since cefazolin does not cover for *Enterococcus* species and most of the Gram-negative rods [26]. Broadening the prophylaxis to cefuroxime combined with vancomycin or teicoplanin may be an option. Previous studies compared the efficacy of various antibiotic prophylaxis regimens in reducing infection rates after joint arthroplasty, mostly by adding an antibiotic agent with a broader Gram-positive spectrum (such as a glycopeptide). Two studies compared the efficacy of cefazolin with a combined regimen of cefazolin and vancomycin [27, 28]. Liu et al. found a reduction in infections in the combined regimen group [27], while Sewick et al. did not detect a difference between the regimens [28]. Other studies compared the use of cefuroxime and a combined regimen of cefuroxime and teicoplanin and found a reduced infection rate in the combined treatment group [29, 30]. The higher efficacy of the combined regimens in these studies was mainly based on a reduction in infections caused by methicillin-resistant *Staphylococcus aureus*.

Tornero et al. studied the efficacy of dual prophylaxis (cefuroxime plus teicoplanin) and found a reduction in PJI rate solely in obese patients when adding teicoplanin to the antimicrobial regimen [30]. This reduction was mainly observed for PJI due to methicillin-susceptible *Staphylococcus aureus*, which supports the hypothesis that the dosage of antimicrobial prophylaxis may be insufficient in obese patients, since methicillin-susceptible *Staphylococcus aureus* should be fully covered by cefuroxime. A randomized controlled trial is needed to provide conclusive scientific evidence regarding the most effective antibiotic prophylaxis regimen for obese patients. This randomized trial should compare different antibiotic regimens and should show whether the absolute percentage of PJIs indeed decreases when adapting the antibiotic prophylaxis regimen in obese patients receiving total hip arthroplasty.

There are several limitations to our study. First of all, we merely included patients with early PJI treated with DAIR. We did not include chronic infections, which could have provided additional insights, as these patients may have different microbiological profiles. We did not collect data on the total number of joint arthroplasties implanted in the participating hospitals during the study period. Therefore, we do not know the exact incidence of PJI in our cohort. However, previous studies clearly demonstrated a higher rate of PJI in obese patients [6, 7], and therefore, it is likely that implementing the proposed prevention strategies will result in an absolute reduction of PJIs in the obese population. A second limitation is the retrospective design of this study. However, due to thorough recording of clinical and microbiological findings in the electronic patient files of patients with PJI there were few missing variables, by which the results are representative for the included patients. Finally, we did not collect data on the exact disinfective agent that has been applied prior to surgery, because we could not reliably collect the allergies of included patients. However, in consultation with the orthopaedic surgeons in the participating hospitals we are confident that the disinfection procedure has been carried out correctly in each patient, whether with a povidone-alcohol solution or with a chlorhexidine-alcohol solution.

A strength of this study is that we were able to select a large homogenous group of patients with early PJI. Our initial statistical analyses showed that non-obese patients had a significantly higher rate of arthroplasties indicated for fracture than obese patients (27.6% vs 10.6%, p<0.001, S1 File). Since literature indicates that fracture-related PJIs have a lower rate of Gram-negative infections and a higher failure rate compared with primary PJIs [31], we decided to only analyze patients with primary arthroplasties indicated for osteoarthritis.

In conclusion, obese and severely obese patients with early periprosthetic hip infections have higher rates of polymicrobial infections with involvement of enterococci and Gram-negative rods, which emphasizes the importance of improving preventive strategies in this specific patient category.

## Supporting information

S1 File SPSS database. (SAV)
